# Can tryptophan supplement intake at breakfast enhance melatonin secretion at night?

**DOI:** 10.1186/s40101-017-0135-9

**Published:** 2017-02-28

**Authors:** Shunsuke Nagashima, Makoto Yamashita, Chiaki Tojo, Masayuki Kondo, Takeshi Morita, Tomoko Wakamura

**Affiliations:** 1 Graduate School of Medicine, Kyoto University, Kyoto, Japan; 2 Japan Society for the Promotion of Science, Tokyo, Japan; 3 Comprehensive Housing R&D Institute, Sekisui House, Ltd., Kyoto, Japan; 4 Department of Environmental Science, Fukuoka Women’s University, Fukuoka, Japan

**Keywords:** Tryptophan, Bright light exposure, Breakfast, Melatonin, Dim light melatonin onset

## Abstract

**Background:**

Tryptophan (TRP) is an essential amino acid, and it has been suggested that TRP intake at breakfast combined with daytime bright light exposure can increase nocturnal melatonin secretion. However, the mechanisms involved are not yet clear. The aim of this study was to examine the effect of TRP supplement intake at breakfast on nocturnal melatonin secretion under different daytime light intensities in humans.

**Method:**

Twelve subjects (aged 21.3 ± 3.0 years, mean ± standard deviation) participated in a random order in experimental sessions lasting 3 days under four conditions in a laboratory setting. The four conditions were TRP*Bright, Placebo*Bright, TRP*Dim, and Placebo*Dim. A TRP capsule (1000 mg) or a placebo starch capsule (1000 mg) were taken at breakfast. In addition, during the daytime (07:00–18:00), the subjects were asked to stay under different light intensities: >5000 lx (bright) or <50 lx (dim). Saliva samples were collected for measuring the concentration of melatonin. The time courses of melatonin concentration and dim light melatonin onset (DLMO) were compared among the four conditions using repeated measurements analysis of variance (ANOVA).

**Result:**

Nocturnal melatonin concentrations in the bright light condition tended to be higher than in the dim light condition (main effect of light: *p* = .099). Moreover, in the bright light condition, the change in DLMO between baseline and after the intervention was significantly higher than that in the dim light condition (main effect of light: *p* <.001). However, the ANOVA results indicated no significant effect of TRP intake on melatonin secretion.

**Conclusion:**

Our findings indicated that intake of 1000 mg of TRP at breakfast on 1 day did not change nocturnal melatonin secretion, even though TRP is the precursor of melatonin. In contrast, daytime bright light exposure increased nocturnal melatonin secretion and advanced the phase of melatonin onset. Therefore, TRP supplementation, unlike exposure to daytime bright light, does not acutely affect biological rhythm and sleep in humans.

**Trial registration:**

UMIN Clinical Trial Registry: UMIN000024121

**Electronic supplementary material:**

The online version of this article (doi:10.1186/s40101-017-0135-9) contains supplementary material, which is available to authorized users.

## Background

Tryptophan (TRP) is an essential amino acid in humans. Humans cannot produce TRP sufficiently by themselves, so this amino acid must be absorbed in the small intestine from protein-rich foods (for example, milk, eggs, meat, and beans). TRP is metabolized into melatonin via the serotonin pathway. Melatonin, a hormone produced by the pineal gland at night, regulates circadian rhythms and sleep–wake cycles in humans. Therefore, several previous studies have suggested that TRP intake at night could improve nocturnal sleep quality in humans [1]. However, it is not clear when TRP is metabolized to form the melatonin that is secreted by the pineal gland at night.

Wada et al. [2] reported the effect of a TRP-rich breakfast in a field study. Their study indicated that salivary melatonin secretion at 23:00 was increased by consumption of a TRP-rich breakfast with exposure to light of low color temperature during the night, compared with no intervention. Furthermore, a laboratory-based study by Fukushige et al. [3] reported that melatonin secretion at night was significantly increased by consumption of a TRP-rich breakfast with daytime bright light exposure for 3 days, although the secretion was not significantly changed by consumption of a TRP-poor breakfast. Both field and laboratory-based studies indicated that melatonin secretion at night might be increased by TRP intake in the morning if there is exposure to bright light during the daytime. However, these studies had some limitations, for example, a lack of control of daily nutrition and the duration of daily light exposure (which was dependent upon the amount of sunlight).

For melatonin synthesis in the brain, TRP must be transported into the brain through the blood–brain barrier (BBB). When TRP passes through the BBB, it competes with large neutral amino acids (LNAAs: isoleucine, leucine, phenylalanine, tyrosine, and valine). Therefore, a higher plasma TRP/LNAA ratio (mol/mol) is favorable for TRP transportation through the BBB. The plasma TRP/LNAA ratio has been used as an indicator of serotonin synthesis in the brain in both animal and human studies [4–7]. This ratio depends on the contents of a meal; it is increased by intake of TRP-rich protein [7] and carbohydrates [8–10] and decreased by intake of LNAA-rich protein. In Fukushige et al.’s study [3], the TRP/LNAA ratios of both TRP-rich and TRP-poor foods were very similar; therefore, it was difficult to conclude that the increase in melatonin secretion at night was due to the TRP-rich breakfast alone. It is necessary to control the intakes of other nutrients in order to elucidate the effect of TRP intake in the morning on nocturnal melatonin secretion. For the same reason, although TRP intake did not significantly advance subjects’ circadian phases in the study by Fukushige et al. [3], their results were not clear. Nakade et al. [11] reported a positive correlation between the estimated amount of TRP intake at breakfast and Morningness-Eveningness Questionnaire (MEQ) score in children, suggesting that children consuming a higher amount of TRP at breakfast may have an earlier circadian acrophase. Thus, further examination of whether melatonin onset can be advanced by TRP intake at breakfast is needed.

The aim of this study was to investigate if TRP supplement intake at breakfast could enhance nocturnal melatonin secretion, after adjusting for light exposure and intakes of TRP, LNAA, and other nutrients. In this study, we compared the profiles of nocturnal melatonin secretion between individuals taking a TRP or placebo supplement at breakfast.

## Methods

### Subjects and experimental conditions

The subjects were male university students recruited voluntarily using a website and posters. None of the subjects had a history of night shift working, tobacco smoking, taking medications, food allergy, or traveling across time zones for a month prior to the experiment. The participants who had mental or physical disorders (determined by the Cornell Medical Index, CMI [12, 13]), sleep disorders (determined by the Pittsburgh Sleep Quality Index, PSQI [14, 15]), or who were extreme morning or evening types (determined by the MEQ [16, 17]) were excluded from the study. Initially, 14 subjects participated in the experiment; however, only 12 of these (aged 21.3 ± 3.0 years, mean ± standard deviation, range 18–26 years; body mass index 21.8 ± 2.6 kg/m^2^, range 18.5–26.1 kg/m^2^) were able to complete the experimental protocol.

All subjects participated in four conditions, undertaken in a random order, with more than a week between each condition. The four conditions were as follows. TRP*Bright condition, TRP supplement (1000 mg) at breakfast (at 08:00) with bright light exposure (>5000 lx at eye level) during the daytime (between 07:00 and 18:00); Placebo*Bright condition, placebo supplement (starch, 1000 mg) at breakfast with daytime bright light exposure; TRP*Dim condition, TRP supplement at breakfast with daytime dim light exposure (<50 lx); and Placebo*Dim condition, placebo supplement at breakfast with daytime dim light exposure.

The amount of TRP intake at breakfast was based on previous studies. Schneider-Helmert and Spinweber [18] concluded that L-TRP should be administered at a dose of more than 1000 mg to obtain reliable effects on sleep, and Hiratsuka et al. [19] reported that the addition of 1000 to 5000 mg of TRP per day (332 to 1660 mg per meal) had no adverse effects in healthy Japanese women. Accordingly, the participants ingested 1000 mg of TRP at breakfast in the two TRP-containing conditions. With respect to the light condition, Fukushige et al. [3] reported that the pattern of TRP metabolism differed depending on the intensity of daytime light. In the present study, we examined the patterns of TRP metabolism under both bright (>5000 lx) and dim (<50 lx) light conditions.

### Experimental protocol

**Fig. 1:**
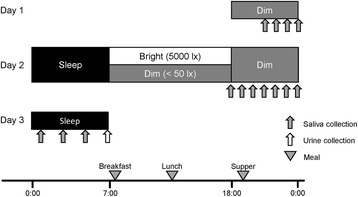
The experimental protocol. *Horizontal bars* indicate experimental light conditions; *gray*, *black*, and *white* represent dim (<50 lx), dark (0 lx), and bright (>5000 lx) lights, respectively. *Arrows* indicate the times of data collection; *gray* and *white arrows* indicate saliva and urine collection times, respectively. *Gray triangles* show the times of meals

The experimental protocol is illustrated in Fig. 1. The subjects were asked to keep to a regular sleep–wake cycle (going to bed at 24:00 and waking up at 07:00) for 3 days prior to the experiment. Throughout this period, adherence to these requirements was confirmed by objective measurements of activity with an Actiwatch (Mini-Mitter Co. Inc., OR, USA).

This experiment was carried out in a living facility consisting of several rooms, one of which each subject stayed in throughout the experiment. Each room was equipped with a bed, table, toilet, and bathroom. The room temperature was controlled within a narrow range (25 ± 2 °C). The subjects were asked to wear a short-sleeved T-shirt and long pants during the experiment. In the experimental rooms, all light sources and windows were covered by black cellophane and black panels, respectively, to create a dim condition. In addition, all curtains were closed in the dim light condition. In this condition, the intensity of the light was <50 lx at eye level when the subject was standing.

On the first day of the experiment, the subjects entered the experimental room with a dim light environment at 18:00. They then took a shower and ate supper in the experimental room. Salivary samples were collected every hour between 21:00 and 24:00 under dim light. After collection of the last saliva sample, they went to bed in the dark (0 lx) at 24:00.

On the second day, the subjects woke at 07:00 and those in the TRP*Dim or Placebo*Dim condition remained in dim light between 07:00 and 18:00. In the TRP*Bright or Placebo*Bright conditions, the subjects stayed in natural sunlight by opening the curtains and taking off the window and light coverings. Moreover, a lighting device (Bright Light Me, Solartone Ltd., Tokyo, Japan) was placed in front of them. A light intensity of >5000 lx was measured at eye level when subjects were sitting in front of the lighting device. The subjects were instructed to spend their free time in front of this device. From 18:00 onwards, all subjects lived in the dim light condition. Breakfast, lunch, and supper were served at 07:30, 12:00, and 19:00, respectively. The subjects were required to eat each meal within a 30-min period. The estimated nutritional values of each meal are shown in Table 1. The nutritional values were based on the Standard Tables of Food Composition in Japan [20]. The capsule (L-tryptophan, 1000 mg: Source Naturals, Inc., CA, USA; or placebo, starch 1000 mg: Maeda Co., Ltd, Ehime, Japan) was taken at 08:00, immediately after breakfast. On the second day, saliva samples were collected every hour between 18:00 and 24:00. After the last saliva sample, the subjects emptied their bladder and went to bed. During sleep time, the subjects were woken to collect saliva samples at 01:00, 03:00, and 05:00 in the dark (0 lx). On the morning of the third day, after collecting a urine sample at 07:00, the subjects were free to leave the experiment. If a subject wanted to pass urine from 24:00 to 07:00, the urine samples were collected into a bottle until 07:00 and acidified with the addition of hydrochloric acid. They were allowed to drink water freely, to read books, and to listen to music throughout the experiment. However, excessive physical exercise, taking a nap, watching TV, and using a mobile phone or computer were not permitted.

**Table 1:** Energy and nutrient intakes at breakfast, lunch, and supper

Energy and nutrients		Breakfast (TRP/placebo)	Lunch	Supper
Energy	(kJ)	1582	3406	4004
Tryptophan	(mg)	1051.9/51.9	307	300
Protein	(g)	4.3	26.1	27.1
Lipid	(g)	7.9	15.6	29.3
Carbohydrate	(g)	72.3	139.5	143.0
Isoleucine	(mg)	154.0	370.4	1064.3
Leucine	(mg)	296.5	684.6	1887.1
Phenylalanine	(mg)	185.7	453.1	1083.7
Tyrosine	(mg)	137.6	319.8	814.7
Valine	(mg)	207.9	503.1	1249.2
LNAA	(mg)	981.7	2330.9	6099.0
TRP/LNAA ratio	(mol/mol)	0.727/0.036	0.089	0.033

Energy and nutrient contents of each meal were calculated from the Standard Tables of Food Composition in Japan [20]

*LNAA* large neutral amino acids (isoleucine, leucine, phenylalanine, tyrosine, valine), *TRP* tryptophan

### Measurements

In this study, salivary melatonin concentrations and creatinine-adjusted urinary melatonin concentrations were measured as indicators of the nocturnal secretion of melatonin. Dim light melatonin onset (DLMO) was calculated from the profile of salivary melatonin concentrations to assess the phase of nocturnal melatonin secretion. DLMO is often used to assess the phase of biological rhythms [21]; in this study, the time of the DLMO was determined by linear interpolation between the time points before and after the melatonin concentration increased and stayed above the 3.3 pg/ml threshold [22, 23].

The saliva samples from all subjects were placed in collection tubes using a pure cotton swab (Sarstedt AG & Co., Nümbrecht, Germany). All salivary samples were immediately centrifuged at 1500*g* for 5 min and frozen at −20 °C until analysis. The urine samples were also stored at −20 °C. The saliva samples were analyzed for melatonin using RIA kits (RK-DSM2 200 tests, Bühlmann Laboratories AG, Schönenbuch, Switzerland). The mean inter-assay and intra-assay coefficients of variation (CV) were 7.9 and 9.8%, respectively. The limit of detection (LoD) was 0.2 pg/ml, and the limit of quantification (LoQ) was 0.9 pg/ml.

The urine samples were analyzed for melatonin with RIA kits (RK-MEL2 200 tests, Bühlmann Laboratories AG, Schönenbuch, Switzerland). The mean intra- and inter-assay CVs were 7.9 and 11.7%, respectively. The LoD was 0.3 pg/ml, and the LoQ was 0.9 pg/ml. Creatinine was analyzed using creatinine kits (Determiner L CRE, Kyowa Medex Co. Ltd., Tokyo, Japan). The mean intra- and inter-assay CVs were <5 and <3%, respectively. The LoD was 0.04 mg/dl, and the LoQ was 0.1 mg/dl. All samples were measured by SRL Co. Ltd. (Tokyo, Japan).

### Statistical analysis

Two participants did not complete the experimental protocol, so data from 12 participants were analyzed. In this study, we compared the time courses of salivary melatonin concentration (from the second to the third day), DLMO, and creatinine-adjusted urine melatonin concentration among the four conditions.

The time courses of salivary melatonin concentration were analyzed by three-way repeated measures ANOVA analysis of variance (ANOVA); TRP (TRP vs. placebo intake), light (bright light vs. dim light), and time of day were the within-subject factors. Creatinine-adjusted urine melatonin concentrations measured in the morning of the third day were compared among conditions by using two-way repeated measures ANOVA. DLMOs were compared between the first and second day in each condition using paired *t* tests. The change in DLMO was compared among the four conditions using two-way repeated measures ANOVA; TRP/placebo and light intensity were the within-subject factors. For all ANOVAs, if the sphericity assumption was not met, the degrees of freedom were modified using the Greenhouse–Geisser method. When any main effects and interactions were significant, the Bonferroni method was used for multiple comparisons.

Data are provided as the mean ± standard error of the mean (SEM), and effect sizes are described with *p* values. Cohen’s *dz* was used as the effect size in paired *t* tests and multiple comparison of ANOVA, and partial *η* ^*2*^ was also used in ANOVA. The sample size was *n* = 12, which was calculated by the effect size as follows: *dz* = 1.2 from the result of DLMO in a previous study [24], alpha error = .0083 (alpha error was modified from .05 for multiple comparisons between the four conditions using the Bonferroni method), and beta error = .80 on the two-tailed test. Statistical analyses were performed using SPSS ver. 21 (IBM, Tokyo, Japan), and a *p* value of <.05 was considered to be significant.

### Ethical considerations

The subjects received an explanation of the research purpose and the experimental protocol from the chief investigator, and informed consent was obtained from each subject before participating in this experiment. This experimental protocol was conducted in accordance with the Declaration of Helsinki and approved by the Ethics Committee at Fukuoka Women’s University.

## Results

### The time course of nocturnal melatonin concentration

**Fig. 2:**
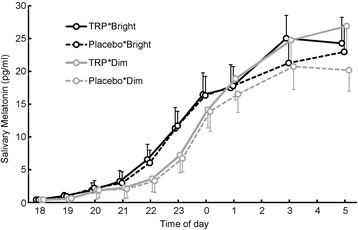
The time courses of salivary melatonin secretion in the four conditions. Bright light and dim light conditions are indicated by the *black line* and *gray line*, respectively. TRP supplementation and placebo supplementation conditions are illustrated by *solid* and *dotted lines*, respectively. The *error bars* indicate the standard errors of the means

The time courses of nocturnal saliva melatonin concentration are illustrated in Fig. 2, and the results from the three-way repeated measures ANOVA are shown in Table 2. There was no significant main effect of TRP intake (*p* = .139, *F*_(1, 11)_ = 2.55, partial *η* ^2^ = .188), and no interactions were significant. Only the main effect of time was significant (*p* <.001, *F*_(1.81, 19.93)_ = 25.62, partial *η* ^2^ = .700), and there was a trend towards a significant main effect of light (*p* = .099, *F*_(1, 11)_ = 3.25, partial *η* ^2^ = .228).

**Table 2:** Analysis of variance results for the time courses of salivary melatonin secretion

	*p* value	*F* value_(df)_	Partial *η* ^2^ value
Main effect of TRP	.139	2.55_(1, 11)_	.188
Main effect of light	.099	3.25_(1, 11)_	.228
Main effect of time	<.001	25.62_(1.81, 19.93)_^a^	.700
Interaction between TRP and light	.391	0.80_(1, 11)_	.068
Interaction between TRP and time	.111	2.49_(1.85, 20.36)_^a^	.184
Interaction between light and time	.118	2.09_(3.08, 33.90)_^a^	.160
Interactions between TRP, light, and time	.342	1.16_(4.05, 44.54)_^a^	.095

^a^The degrees of freedom were modified using the Greenhouse–Geisser method because the assumption of sphericity was not met

*TRP* tryptophan

### Dim light melatonin onset

The DLMOs are shown in Table 3. Since the DLMOs of four subjects could not be assessed during the sampling period (their melatonin levels did not rise above the absolute threshold (3.3 pg/ml)), their data were excluded from the analysis. The DLMOs on the second day were significantly earlier than those on the first day in the TRP*Bright and Placebo*Bright conditions (*p* = .001, *dz* = 1.81 and *p* = .002, *dz* = 1.73, respectively). However, in the TRP*Dim and Placebo*Dim conditions, there was no significant difference between the DLMOs on the first and second days (TRP*Dim condition: *p* = .050, *dz* = 0.84; and Placebo*Dim: *p* = .118, *dz* = 0.63, respectively). Regarding changes in DLMO (Tables 3 and 4), two-way repeated measures ANOVA showed a significant main effect of light (*p* <.001, *F*_(1, 7)_ = 42.03, partial *η* ^*2*^ = .857). Post hoc tests indicated the change in DLMO in the bright light condition was significantly greater than that in the dim light condition. The main effect of TRP and the interaction between TRP and light were not significant.

**Table 3:** DLMOs in the four conditions (*n* = 8)

	Day 1 DLMO (h:min)	Day 2 DLMO (h:min)	*p* value	*t* value	*dz* value	Δ DLMO^a^ (min)
TRP*Bright	22:51 ± 0:11	21:53 ± 0:17	.001	5.12	1.81	+58.5 ± 11.4
Placebo*Bright	22:26 ± 0:12	21:40 ± 0:15	.002	4.89	1.73	+46.6 ± 9.5
TRP*Dim	22:01 ± 0:09	22:24 ± 0:14	.050	2.37	0.84	−22.7 ± 9.6
Placebo*Dim	22:21 ± 0:12	22:35 ± 0:17	.118	1.78	0.63	−13.8 ± 7.8

A DLMO was not detected for four subjects during the sampling period; therefore, their data were excluded from this analysis. All data are shown as the mean ± standard error of the mean. Times for day 1 DLMO and day 2 DLMO are presented as clock time (h:min). Δ DLMO = change in DLMO (min)

*DLMO* dim light melatonin onset

^a^A positive value for Δ means that the DLMO on the second day had advanced from its value on the first day

**Table 4:** The repeated measures analysis of variance results for change in dim light melatonin onset

	*p* value	*F* value_(*df*)_	Partial *η* ^2^ value
Main effect of TRP	.880	0.03 _(1, 7)_	.004
Main effect of light	<.001	42.03 _(1, 7)_	.857
Interaction between TRP and light	.115	3.23 _(1, 7)_	.316

*TRP* tryptophan

### Creatinine-adjusted urine melatonin

**Fig. 3:**
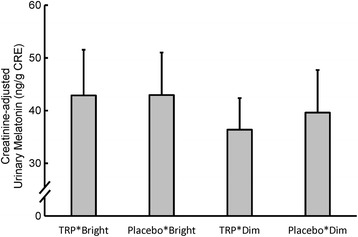
Creatinine-adjusted urinary melatonin in the four conditions (*n* = 12). The *error bars* indicate the standard errors of the means

The creatinine-adjusted melatonin concentrations in urine on the morning of the third day are illustrated in Fig. 3. Two-way repeated measures ANOVA indicated that the values in the bright light conditions were significantly higher than those in the dim light conditions (*p* = .038, *F*_(1, 11)_ = 0.29, partial *η* ^*2*^ = .026). However, TRP supplementation had no significant effect (*p* = .599, *F*_(1, 11)_ = 5.53, partial *η* ^*2*^ = .335), and the interaction between TRP and light was also not significant (*p* = .730, *F*_(1, 11)_ = 0.13, partial *η* ^2^ = .011).

## Discussion

Our results showed that nocturnal melatonin secretion was increased by exposure to bright light in the daytime after only 1 day. This result is supported by several previous studies. Hashimoto et al. [25] reported that exposure to bright light (~5000 lx) in the daytime for three consecutive days significantly advanced the onset of nocturnal melatonin secretion in young people. In addition, Takasu et al. [26] reported that the amplitude of nocturnal melatonin secretion was higher when subjects were exposed to daytime bright light for 7 days than when they were exposed to dim light for the same duration. Furthermore, other studies have indicated that daytime bright light exposure could prevent melatonin suppression induced by nighttime light [27, 28]. Therefore, in the dim light conditions of our experiment, it is possible that nocturnal melatonin suppression may have been due to exposure to a light intensity of less than 50 lx.

The effects of bright light exposure were very strong and their effect sizes were very large (*dz* = 1.97 after TRP supplementation and *dz* = 2.21 after placebo supplementation; see Additional file 1A). The advance in the phase of nocturnal melatonin secretion after daytime exposure to bright light was consistent with several previous studies [25, 29, 30]. A previous experimental study, which used an isolation unit without temporal information, indicated that the sleep–wake cycle and the rhythm of the core body temperature were advanced by a single exposure to bright light (of 3- or 6-h duration) given early in the day (in the morning) [31]. Our findings also suggest that 1 day of diurnal bright light exposure might be effective in advancing delayed biological rhythms, such as those found in delayed sleep phase syndrome and some circadian and sleep disorders, in real life.

One aim of our study was to investigate whether TRP supplementation at breakfast increased the secretion of melatonin at night and/or advanced the phase of melatonin secretion. However, a TRP supplementation of 1000 mg at breakfast for 1 day did not exert such effects. The results of the present study were inconsistent with those of Fukushige et al. [3] and with other studies suggesting that TRP intake might improve human sleep by increasing nocturnal melatonin secretion [2, 11, 32]. This disagreement may have arisen from the following four reasons.

First, the period of TRP intake in our study was different from that in the study by Fukushige et al. [3]. Our participants took 1000 mg of TRP at breakfast for only 1 day, whereas in the study by Fukushige et al., a TRP-rich breakfast (including 476 mg of TRP) was eaten for three consecutive days. This suggests that TRP ingested in food might need a period of more than 1 day to be converted into melatonin. This suggestion is supported by a study of the circadian rhythms of serum TRP and blood serotonin in humans. Rao et al. [33] reported that the acrophase of the circadian rhythm of the blood serotonin levels was earlier by 5.1–6.5 h than that of the serum TRP levels in both schizophrenic and healthy humans. TRP is a precursor of serotonin; therefore, it is reasonable to suppose that TRP would be metabolized into serotonin for more than 17.5 h. Accordingly, if the duration of TRP supplementation was longer than in our study, its effect might have been greater than in the placebo control condition.

Second, intake of other nutrients affects TRP metabolism. TRP needs several enzymes to be converted into melatonin [34]. Aromatic L-amino acid decarboxylase and arylalkylamine *N*-acetyltransferase are two of the enzymes involved in serotonin and melatonin synthesis, and these enzymes are influenced by the intake of other nutrients, including vitamin B6 and *n*-3 fatty acids. That is, not only TRP intake but also vitamin B6 and *n*-3 fatty acid intake might need to be increased in order to convert TRP into melatonin in the brain, even if the TRP consumed at breakfast has passed through the BBB. Therefore, it is possible that the combination of TRP supplementation with the intake of other nutrients in the morning would more effectively increase the secretion of melatonin at night.

Third, Yao et al. [35] mentioned in their review that over 95% of ingested TRP is catabolized in the liver via the kynurenine pathway and that only 1–2% of dietary TRP is converted into serotonin in healthy adult humans. Moreover, TRP is converted into serotonin mainly in the small intestine. This means that very little of the serotonin in the brain has been converted from TRP. Therefore, TRP supplementation of 1000 mg might do little to increase nocturnal melatonin secretion. In addition, Schneider-Helmert and Spinweber [18] reviewed studies on the hypnotic efficacy of L-TRP and concluded that it should be administered at a dose of between 1000 and 5000 mg in order to obtain reliable effects on sleep (L-TRP was administered 20 min before bedtime), and severe, chronic insomniacs appeared to respond to doses of L-TRP as low as 1000 mg only after repeated administration. Similarly, Adam and Oswald [36] reported that there was no effect of TRP on the latency to sleep onset. Therefore, TRP supplementation of 1000 mg in the present study may have been too low to increase melatonin secretion. Further studies investigating the effect of higher amounts of TRP supplementation in the morning are needed. However, considering the mechanism of TRP metabolism, taking TRP in order to increase melatonin secretion might not be justified.

Fourth, there were some biases in the present study. As our subjects were healthy male adults, there was a “healthy adult” bias. Previous studies have indicated that TRP intake was significantly effective for patients with insomnia [18, 37]; however, in healthy adults, melatonin secretion at night might be sufficient, so that the level of nocturnal melatonin secretion reaches a peak and would not be raised further by TRP supplementation. Moreover, age may also be associated with TRP metabolism. Harada et al. [32] reported that the amount of TRP intake at breakfast was significantly correlated with the inherited circadian type (morning or evening type) in infants and lower grade elementary school students but that this correlation disappeared in higher grade elementary school students and junior high school students. Their findings suggested that any effects of TRP supplementation in the morning upon nocturnal melatonin secretion might disappear as individuals grow older. That is, TRP intake in the morning might affect nocturnal melatonin secretion only in children.

Our study had some methodological limitations. Creatinine-adjusted urinary melatonin may not have been a suitable outcome measure. Urinary melatonin may still have been secreted at 07:00 because the salivary melatonin concentration appeared to peak at 05:00. Therefore, we could not evaluate the total amount of nocturnal melatonin secretion. Moreover, we collected saliva samples using a cotton swab. A previous study indicated that cotton swabs might decrease melatonin levels [38]. For this reason, we could not determine the DLMO of four subjects, who had melatonin levels lower than 3.3 pg/ml. Further research is needed to investigate other ways of measuring melatonin secretion (e.g., serum melatonin concentration).

## Conclusions

Our study examined whether TRP ingested at breakfast was metabolized in vivo into melatonin at night, adjusting for the biases in Fukushige et al.’s study [3]. However, it was found that 1 day of 1000 mg supplementation with TRP had no significant effect on nocturnal melatonin secretion, even though TRP is the precursor of both serotonin and melatonin. However, daytime exposure to bright light increased nocturnal melatonin secretion and advanced the time of DLMO. These results suggest that a single TRP supplement would not acutely promote sleep or alter the timing of biological rhythms in humans, unlike the effects of exposure to bright light in the daytime. In recent years, we have encountered more and more commercial articles insisting that TRP intake could improve sleep by increasing nocturnal melatonin secretion. However, these articles lack scientific evidence and should not be accepted without question. These articles fail to pay attention to the mechanisms by which TRP is metabolized. In order to investigate the possibility that TRP supplementation improves sleep quality or affects the timing of circadian rhythms, further studies are needed to examine the correlation between TRP metabolism and nocturnal melatonin secretion.

## Additional file

Additional file 1: The results of multiple comparisons of ∆ DLMO. (DOCX 16 kb)

## Abbreviations

ANOVA: Analysis of variance
BBB: Blood–brain barrier
CMI: Cornell Medical Index
CV: Coefficient of variation
DLMO: Dim light melatonin onset
LNAA: Large neutral amino acid
LoD: Limit of detection
LoQ: Limit of quantification
MEQ: Morningness-Eveningness Questionnaire
Placebo*Bright: Placebo supplement intake and bright light environment condition
Placebo*Dim: Placebo supplement intake and dim light environment condition
PSQI: Pittsburgh Sleep Quality Index
TRP: Tryptophan
TRP*Bright: Tryptophan supplement intake and bright light environment condition
TRP*Dim: Tryptophan supplement intake and dim light environment condition

## References

[1] Wyatt RJ, Engelman K, Kupfer DJ, Fram DH, Sjoerdsma A, Snyder F (1970). Effects of L-tryptophan (a natural sedative) on human sleep. Lancet.

[2] Wada K, Yata S, Akimitsu O, Krejci M, Noji T, Nakade M (2013). A tryptophan-rich breakfast and exposure to light with low color temperature at night improve sleep and salivary melatonin level in Japanese students. J Circadian Rhythms.

[3] Fukushige H, Fukuda Y, Tanaka M, Inami K, Wada K, Tsumura Y (2014). Effects of tryptophan-rich breakfast and light exposure during the daytime on melatonin secretion at night. J Physiol Anthropol.

[4] Fernstrom JD, Wurtman RJ (1972). Brain serotonin content: physiological regulation by plasma neutral amino acids. Science.

[5] Markus CR (2007). Effects of carbohydrates on brain tryptophan availability and stress performance. Biol Psychol.

[6] Markus CR (2008). Dietary amino acids and brain serotonin function; implications for stress-related affective changes. Neuromolecular Med.

[7] Markus CR, Verschoor E, Firk C, Kloek J, Gerhardt CC (2010). Effect of tryptophan-rich egg protein hydrolysate on brain tryptophan availability, stress and performance. Clin Nutr.

[8] Lyons PM, Truswell AS (1988). Serotonin precursor influenced by type of carbohydrate meal in healthy adults. Am J Clin Nutr.

[9] Pan RM, Mauron C, Glaeser B, Wurtman RJ (1982). Effect of various oral glucose doses on plasma neutral amino acid levels. Metabolism.

[10] Wurtman RJ, Wurtman JJ, Regan MM, McDermott JM, Tsay RH, Breu JJ (2003). Effects of normal meals rich in carbohydrates or proteins on plasma tryptophan and tyrosine ratios. Am J Clin Nutr.

[11] Nakade M, Akimitsu O, Wada K, Krejci M, Noji T, Taniwaki N (2012). Can breakfast tryptophan and vitamin B6 intake and morning exposure to sunlight promote morning-typology in young children aged 2 to 6 years?. J Physiol Anthropol.

[12] Brodman K, Erdmann AJ, Wolff HG. Cornell Medical Index Health Questionnaire: Manual. New York: Cornell University Medical College; 1949.

[13] Kanehisa T, Fukamachi T (1972). CMI, Cornell Medical Index.

[14] Buysse DJ, Reynolds CF, Monk TH, Berman SR, Kupfer DJ (1989). The Pittsburgh Sleep Quality Index: a new instrument for psychiatric practice and research. Psychiatry Res.

[15] Doi Y, Minowa M, Uchiyama M, Okawa M (1998). Development of the Japanese version of the Pittsburgh Sleep Quality Index. Jpn J Psychiatry Treatment.

[16] Horne JA, Östberg O (1976). A self-assessment questionnaire to determine morningness-eveningness in human circadian rhythms. Int J Chronobiol.

[17] Ishihara K, Saitoh T, Inoue Y, Mikita Y (1984). Validity of the Japanese version of the Morningness-Eveningness Questionnaire. Percept Mot Skills.

[18] Schneider-Helmert D, Spinweber CL (1986). Evaluation of L-tryptophan for treatment of insomnia: a review. Psychopharmacology (Berl).

[19] Hiratsuka C, Fukuwatari T, Sano M, Saito K, Sasaki S, Shibata K (2013). Supplementing healthy women with up to 5.0 g/d of L-tryptophan has no adverse effects. J Nutr.

[20] Japanese Ministry of Education, Culture, Sports, Science and Technology. Standard Tables of Food Composition in Japan -2010-. Tokyo: Ministry of Education, Culture, Sports, Science and Technology; 2010.

[21] Pandi-Perumal SR, Smits M, Spence W, Srinivasan V, Cardinali DP, Lowe AD (2007). Dim light melatonin onset (DLMO): a tool for the analysis of circadian phase in human sleep and chronobiological disorders. Prog Neuropsychopharmacol Biol Psychiatry.

[22] Campbell SS, Murphy PJ (1998). Extraocular circadian phototransduction in humans. Science.

[23] Suzuki H, Uchiyama M, Tagaya H, Ozaki A, Kuriyama K, Aritake S (2004). Dreaming during non-rapid eye movement sleep in the absence of prior rapid eye movement sleep. Sleep.

[24] Revell VL, Kim H, Tseng CY, Crowley SJ, Eastman CI (2005). Circadian phase determined from melatonin profiles is reproducible after 1 wk in subjects who sleep later on weekends. J Pineal Res.

[25] Hashimoto S, Kohsaka M, Nakamura K, Honma H, Honma S, Honma K (1997). Midday exposure to bright light changes the circadian organization of plasma melatonin rhythm in humans. Neurosci Lett.

[26] Takasu NN, Hashimoto S, Yamanaka Y, Tanahashi Y, Yamazaki A, Honma S (2006). Repeated exposures to daytime bright light increase nocturnal melatonin rise and maintain circadian phase in young subjects under fixed sleep schedule. Am J Physiol Regul Integr Comp Physiol.

[27] Hebert M, Martin S, Lee C, Eastman C (2002). The effects of prior light history on the suppression of melatonin by light in humans. J Pineal Res.

[28] Kozaki T, Kubokawa A, Taketomi R, Hatae K (2015). Effect of day-time exposure to different light intensities on light-induced melatonin suppression at night. J Physiol Anthropol.

[29] Kanikowska D, Hirata Y, Hyun K, Tokura H (2001). Acute phase proteins, body temperature and urinary melatonin under the influence of bright and dim light intensities during the daytime. J Physiol Anthropol Appl Human Sci.

[30] Wakamura T, Tokura H (2000). The influence of bright light during the daytime upon circadian rhythm of core temperature and its implications for nocturnal sleep. Nurs Health Sci.

[31] Honma K, Honma S, Wada T (1987). Phase-dependent shift of free-running human circadian rhythms in response to a single bright light pulse. Experientia.

[32] Harada T, Hirotani M, Maeda M, Nomura H, Takeuchi H (2007). Correlation between breakfast tryptophan content and morning-evening in Japanese infants and students aged 0-15 yrs. J Physiol Anthropol.

[33] Rao LM, Gross G, Strebel B, Halaris A, Huber G, Bräunig P (1994). Circadian rhythm of tryptophan, serotonin, melatonin, and pituitary hormones in schizophrenia. Biol Psychiatry.

[34] Peuhkuri K, Sihvola N, Korpela R (2012). Diet promotes sleep duration and quality. Nutr Res.

[35] Yao K, Fang J, Yin YL, Feng ZM, Tang ZR, Wu G (2011). Tryptophan metabolism in animals: important roles in nutrition and health. Front Biosci (Schol Ed).

[36] Adam K, Oswald I (1979). One gram of l-tryptophan fails to alter the time taken to fall asleep. Neurophamacology.

[37] Hudson C, Hudson SP, Hecht T, MacKenzie J (2005). Protein source tryptophan versus pharmaceutical grade tryptophan as an efficacious treatment for chronic insomnia. Nutr Neurosci.

[38] Kozaki T, Lee S, Nishimura T, Katsuura T, Yasukouchi A (2011). Effects of saliva collection using cotton swabs on melatonin enzyme immunoassay. J Circadian Rhythms.

