# Supplementary material for: Can tryptophan supplement intake at breakfast enhance melatonin secretion at night?
Source: J Physiol Anthropol. 2017 Feb 28;36:20. doi: 10.1186/s40101-017-0135-9 (PMC5331733; doi:10.1186/s40101-017-0135-9)
Supplement: Additional file 1: — The results of multiple comparisons of ∆ DLMO. (DOCX 16 kb) [file 40101_2017_135_MOESM1_ESM.docx]

**Additional file 1.docx: The results of multiple comparisons of Δ DLMO.**

**A: The result of multiple comparisons of Δ DLMO (min: *mean* ± *SEM*) between bright light and dim light conditions**

|  | **Bright light** | **Dim light** | **Δ (bright and dim light)** | ***dz*-value** |
| --- | --- | --- | --- | --- |
| TRP supplement condition | +58.5 ± 11.4 | −22.7 ± 9.6 | +81.1 ± 14.5 | 1.97 |
| Placebo supplement condition | +46.6 ± 9.5 | −13.8 ± 7.8 | +60.4 ± 9.7 | 2.21 |

A positive value for Δ means simple phase-advance shift by bright light exposure.

**B: The result of multiple comparisons of Δ DLMO (min: *mean* ± *SEM*) between the TRP and placebo supplement conditions**

|  | **TRP** | **Placebo** | **Δ (TRP and placebo)** | ***dz*-value** |
| --- | --- | --- | --- | --- |
| Bright light condition | +58.5 ± 11.4 | +46.6 ± 9.5 | +11.8 ± 13.0 | 0.32 |
| Dim light condition | −22.7 ± 9.6 | −13.8 ± 7.8 | −8.9 ± 8.6 | 0.36 |

A positive value for Δ means simple phase-advance shift by TRP supplement intake, and a negative value for Δ means simple phase-delay shift by TRP supplement intake.
